# Evaluation of the *CYP1B1* gene as a candidate gene in beagles with primary open-angle glaucoma (POAG)

**Published:** 2009-11-28

**Authors:** K. Kato, A. Kamida, N. Sasaki, B.S. Shastry

**Affiliations:** 1 The laboratory of Veterinary Emergency Medicine and Veterinary Surgery Graduate School of Agricultural and Life Sciences, The University of Tokyo, Tokyo, Japan; 2 Department of Biological Sciences, Oakland University, Rochester, MI

## Abstract

**Purpose:**

In humans, primary open-angle glaucoma (POAG) is a complex genetic disorder and is the leading cause of visual impairment. Although all relevant genes were not identified, a small subset of the condition is found to be caused by mutations in the *MYOC* and *CYP1B1* genes. Inherited glaucoma also occurs in several breeds of dogs including beagles. Primary glaucoma in beagles is inherited as an autosomal recessive trait. The purpose of this study is to investigate the role of the *CYP1B1* gene in beagles with POAG.

**Methods:**

For the purpose of genetic analysis, total RNAs from the spleen of the canines were isolated and *CYP1B1* cDNA was prepared. Genomic DNA from five affected, two carriers, and 13 randomly selected normal beagles with no sign of glaucoma was amplified by the polymerase chain reaction (PCR) using four pairs of primers. The amplified products were directly sequenced using BigDye terminator cycle sequencing.

**Results:**

Genomic DNA analyses have identified a substitution polymorphism (109A→C) in the 5’-untranslated region (UTR) as well as a missense mutation (P93R) in exon 2 of the gene. Three affected, two carriers, and nine normal dogs are heterozygous while two affected and three normal dogs are homozygous for the missense mutation. One normal dog did not show this alteration. Normal dogs also contain the substitution polymorphism in the 5’-UTR. Similar experiments with exon 3 did not identify any additional mutation in the gene.

**Conclusions:**

The above results suggest that *CYP1B1* alterations in the coding and UTR are not the primary cause of glaucoma in beagles by possible monogenic association. They may be classified as polymorphisms or they may modify glaucoma phenotype.

## Introduction

Glaucoma in humans is a complex, genetically heterogeneous condition and is the leading cause of blindness worldwide. It is classified into primary open-angle (POAG), primary closed-angle (PCAG) and primary congenital glaucoma (PCG). The disease is frequently associated with elevated intraocular pressure (IOP) and involves defects in the trabecular meshwork (TM) and the anterior chamber of the eye, leading to the obstruction of aqueous outflow, IOP, progressive degeneration of the optic nerve and retinal ganglion cell death causing blindness mostly in adults [1]. Mechanisms independent of IOP are also implicated in glaucomatous degeneration. Several family studies suggest that specific genetic defects may contribute to the pathogenesis of POAG. Although details about the inheritance of the disease remain unclear, genetic regions that are influencing POAG have been identified in large population based studies. Subsequent studies have demonstrated that mutations in myocilin (*MYOC*) and optineurin (*OPTN*) genes are associated with POAG in various populations [2]. Mutations in *MOYC* and *OPTN* together account for less than 5% of all POAG cases. In addition, mutations in *CYP1B1* – a member of the cytochrome P450 superfamily enzymes – have been implicated in POAG and juvenile open-angle glaucoma [3-5]. In children, PCG is an important cause of visual loss and is diagnosed during the neonatal or infantile period [6-8]. Inheritance of PCG is believed to be autosomal recessive [9]. Recently, three PCG loci (2p21, 1p36, and 14q24.3) have been mapped and a spectrum of mutations in *CYP1B1* (GLC 3A) have been implicated in the pathogenesis of the disorder, suggesting that *CYP1B1* is the major PCG causing gene [10-15]. Mutations in *CYP1B1* are associated with wide range of phenotypes and the alteration of this gene could impair the morphogenesis of the outflow angle because it has been proposed that *CYP1B1* participates in iridocorneal angle development [16]. Therefore, it may play an important role in the physiology and development of the eye. Recently it has been suggested that it may be a potential modifier gene in early onset glaucoma [17].

The biological basis of the disease is not fully understood. Investigations in human glaucoma have been hampered because of a lack of suitable inherited POAG animal models of human disease with the exception of inherited glaucoma in beagles. The condition in beagles is transmitted as an autosomal recessive trait and appears when the animals are nine to 18 months old [18]. The pathogenesis, clinical signs, and pharmacological responses of the glaucoma in beagles have been previously investigated and reported [18-20]. For our knowledge, this model system has not been investigated at the genetic level. Therefore, it is of interest for us to conduct genetic analysis on this only available beagle model of open-angle glaucoma. Such studies may provide additional insight about the mechanism of this disease and also complement the human studies.

## Methods

### Isolation of canine *CYP1B1* cDNA

For the purpose of genetic studies, canine *CYP1B1* cDNA was isolated. Three normal beagles were euthanized with an intravenous overdose of pentobarbital sodium (these procedures were approved by the animal care and use committee of the graduate school of agriculture and life sciences, University of Tokyo, Japan) and small pieces of spleen tissues were excised. By using an RN easy mini-kit (Qiagen, Tokyo, Japan) total RNAs were extracted from the tissue. From this total RNAs, single stranded cDNA was synthesized with an oligo(dT)_12-18_ primer and the SuperScript III reverse transcriptase (Invitrogen, Tokyo, Japan). The extracted cDNA was stored at -20 °C. For the synthesis of the second strand we have designed the oligonucleotide primers (Table 1) using the sequence information of the *Canis familiaris* chromosome 17 whole genome shotgun sequence (GenBank). Amplification was carried out according to the procedure supplied by the manufacturer using these primers (Table 1) and Phusion high fidelity DNA polymerase (Finnzymes, Espoo, Finland). Briefly, polymerase chain reaction (PCR) amplification was carried out in a thermocycler (Bio-Rad, Tokyo, Japan) and consisted of an initial denaturation at 98 °C for 30 s, 35 cycles of denaturation at 98 °C for 10 s, annealing at 73 °C for 30 s, and extension at 72 °C for 45 s followed by a final extension at 72 °C for 10 min. The PCR products were electrophoresed on a 2% agarose gel and bands were visualized by ethidium bromide staining. The amplified products were purified by SUPREC-PCR (TaKaRa Bio Inc., Shiga, Japan) or Wizard SV gel and PCR clean up system (Promega, WI).

**Table 1:** Primers used in cDNA isolation and mutational analysis.

**Region**	**5’–3’ Forward**	**5’–3’ Reverse**
5’-UTR	TCCCCTCTCACTGCCTACCA	GGGATGGCGCGTTTTGACTC
Exon 2	CGCGATCTCAGGTTTGCGA	TCATGAGCGCCGTGTGCTT
	TGCGGTTGAGCTGCTCGAA	TCAAAGGCCCCAGCCCTATTACGGA
Nested	AAGCACACGGCGCTCATGACGTT	CACCTCGCAGGTCAGTCTGT
Exon 3	ACACGCATGAAGCACATC	GACTTACGATAGGAAAGAGAGCA

In the table, UTR refers non-translated region (Exon 1).

### DNA sequencing and mutational analysis

The purified product was directly sequenced using a BigDye terminator v 1.1 cycle sequencing kit (Applied Biosystems, Foster city, CA) and an ABI PRISM 3130-Avant genetic analyzer (Applied Biosystems). This sequence information was deposited in GenBank (Accession # FJ 824086.1). The sequence data were aligned and compared by using a genetic information processing software (GENETYX, version 6.1.1.0, Genetyx, Tokyo, Japan) and a sequence scanner software version 1.0 (Applied Biosystems 2005). For mutational analyses, genomic DNA from peripheral blood samples of normal and affected dog was extracted by using an EZ1 – DNA blood kit and the BIOROBOT EZ1 system (Qiagen). Genomic DNA from normal and affected dogs was amplified and subjected to sequence analysis as detailed above by using the same primers (except the nested primer) listed in the Table 1. All three exons were amplified and both strands were sequenced. Sequencing reactions were repeated at least twice using freshly amplified DNA.

## Results

**Figure 1:**
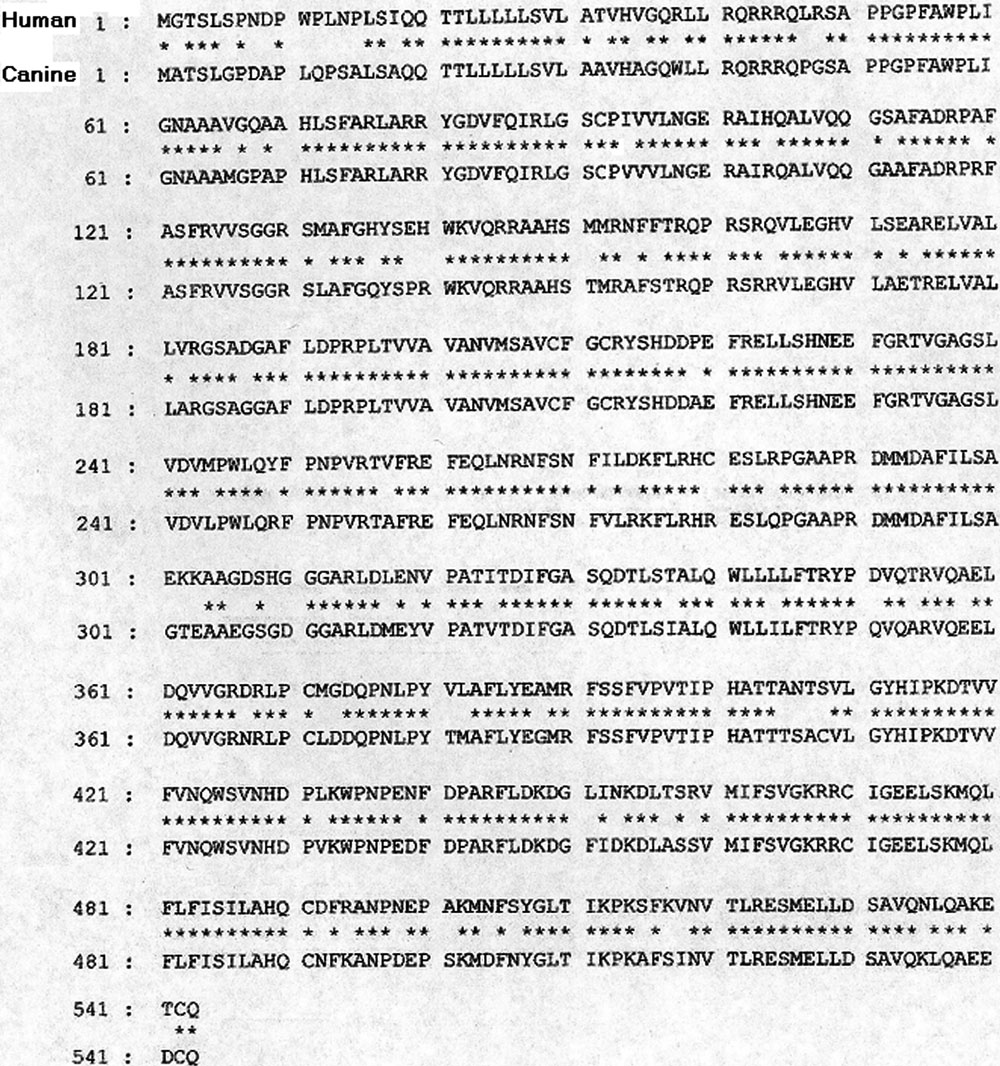
A comparative analysis of amino acid sequence between human and canine *CYP1B1* genes. Canine *CYP1B1* is 84% homologous to human gene at the amino acid and nucleotide level. The asterisk indicates identical amino acids.

**Figure 2:**
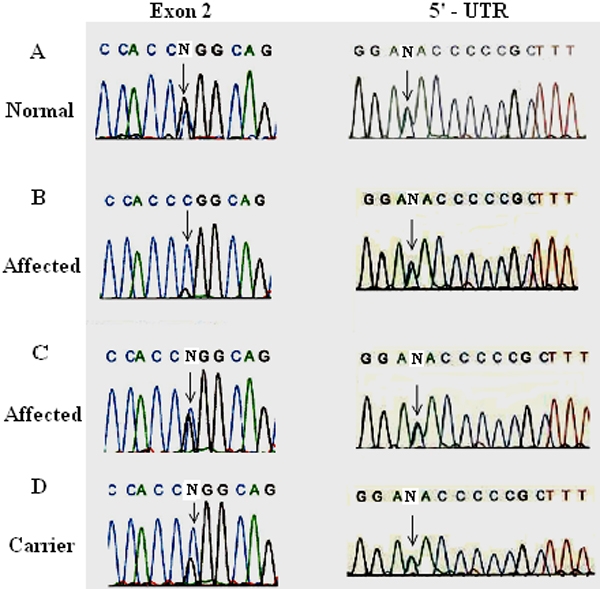
Mutational analysis of *CYP1B1*. Nucleotide sequence of the mutant parts of the exon 2 (left-hand side) and UTR (right hand-side) of CYP1B1 were shown. The nucleotide sequence change in codon 93 is from CCG to CGG (arrows) that resulted in the amino acid arginine (CGG) instead of normal proline (CCG). This missense mutation is present in the normal (heterozygote, **A**), affected (heterozygote, **C**), carrier (heterozygote, **D**) and also in two affected and three normal dogs (homozygote, **B**). The substitution polymorphism in the 5’-UTR (arrows) is present in all animals tested (right-hand side).

In order to understand the genetic basis of POAG in beagles, we have previously shown that the condition is not associated with *MYOC* [21]. As part of our continued interest in POAG, in this report we have isolated canine *CYP1B1* cDNA for mutational analysis. The gene is partially characterized (promoter region and splice sites are not characterized) and contains three exons of which only exons two and three code for the protein while the first exon contains a 5’-untranslated region (UTR) of 354 base pairs. The putative open reading frame begins in the second exon and the gene encodes a 543 amino acid protein. It is approximately 84% homologous to the human gene at the levels of nucleotides and amino acids (Figure 1). For the purpose of genetic analysis, we have analyzed five affected, two carriers, and 13 randomly selected normal beagles with no sign of glaucoma. The results are summarized in Table 2. Our analyses have identified a substitution polymorphism (109A→C) in the 5’-UTR (Figure 2A-D, right-hand side) and it is present in affected, carrier and normal dogs (Figre 2, right-hand side). Additionally, a heterozygous missense mutation (P93R) in exon 2 of the gene was identified in three affected and two carrier dogs (Figure 2C,D, left-hand side) while two affected dogs are homozygous (Figure 2B, left-hand side) for this mutation. Among normal animals, nine of thirteen revealed heterozygosity for the missense mutation (Figure 2A, left-hand side), three of them are homozygous and one normal dog did not contain this alteration. The missense mutation has changed the encoded amino acid proline of the codon 93 to arginine (P93R). Similar experiments with exon 3 did not identify any additional mutation in the gene.

**Table 2:** Polymorphic changes identified in *CYP1B1* in beagles.

**Analysis of canine samples**	**Exon 2**		**5’-UTR**	
	**Mutational changes**	**Genotype**	**Polymorphic changes**	**Genotype**
1 normal	P93P	homozygote	109 A→C	heterozygote
9 normal	P93R	heterozygote	ND	—
3 normal	R93R	homozygote	ND	—
3 affected	P93R	heterozygote	109 A→C	heterozygote
2 affected	R93R	homozygote	109 A→C	heterozygote
2 carriers	P93R	heterozygote	109 A→C	heterozygote

The affected dogs have POAG. In the table, ND refers to not determined, P refers to proline (normal amino acid), and R refers to arginine.

## Discussion

Glaucoma in beagles is inherited as an autosomal recessive trait and the genetic basis of the disorder is not known. In this report, we have isolated and analyzed canine CYP1B1 gene (which is also mutated in human disorder) for mutations. Our analyses have identified a substitution polymorphism in the 5’-UTR and a missense mutation in the exon 2 of the gene. First of all, because normal dogs have the same 5’-UTR substitution (heterozygote) and three of the thirteen normal dogs contain the same missense mutation in both alleles (frequency is about 24%) it is likely that these variations are non-pathogenic. Secondly, these alterations did not segregate with the disease. Hence it is also unlikely that it is pathogenic. The amino acid proline at codon 93 is conserved in normal mouse, rat and humans. It is surprising that its alteration to arginine did not produce the phenotype in homozygote animals (proline is considered to be a helix-breaking residue). Therefore, it is tempting to speculate that this mutation may also not have any functional significance or the mutation is hypomorphic similar to the other reported mutations [22]. It is interesting that many (approximately 72%) normal dogs are heterozygous for the missense mutation in the canine population and there are three classes of normal beagles (no mutation, heterozygotes and homozygotes) at the *CYP1B1* locus. Whatever the real explanation for these observed results, glaucoma in beagles does not appear to be a simple recessive trait and it may have a multifactorial etiology. Functional assays as well as mating experiments in the future may clarify the physiological role (if any) of polymorphic missense and substitution mutations. Meanwhile, identification of a putative predisposing gene may substantially improve our knowledge of the pathogenesis of glaucoma in beagles.
